# Raw pet food as a risk factor for shedding of extended-spectrum beta-lactamase-producing *Enterobacteriaceae* in household cats

**DOI:** 10.1371/journal.pone.0187239

**Published:** 2017-11-02

**Authors:** Valérie O. Baede, Els M. Broens, Mirlin P. Spaninks, Arjen J. Timmerman, Haitske Graveland, Jaap A. Wagenaar, Birgitta Duim, Joost Hordijk

**Affiliations:** 1 Department of Infectious Diseases and Immunology, Faculty of Veterinary Medicine, Utrecht University, Utrecht, the Netherlands; 2 Wageningen Bioveterinary Research, Lelystad, the Netherlands; Texas A&M University College Station, UNITED STATES

## Abstract

**Background:**

Close contact between pets and owners provides the opportunity for transmission of antimicrobial resistant organisms like extended-spectrum beta-lactamase (ESBL)/AmpC beta-lactamase (AmpC)-producing *Enterobacteriaceae*, posing a risk to public health.

**Objectives:**

To investigate whether raw feed is a risk factor for household cats to shed ESBL-producing *Enterobacteriaceae*, a cohort study was designed. Additionally, raw and non-raw commercial pet food products were screened for the presence of ESBL-producing *Enterobacteriaceae*.

**Methods:**

Weekly fecal samples of 17 cats in the control group and 19 cats in the exposed group were collected for three weeks and analyzed for the presence of ESBL-producing *Enterobacteriaceae*. Questionnaires were obtained to determine additional risk factors. Fecal samples were cultured on MacConkey agar supplemented with 1 mg/L cefotaxime. PCR and sequence analysis was used for screening for ESBL genes in suspected isolates. Pet food samples were cultured in LB broth supplemented with 1 mg/L cefotaxime and processed as described above.

**Results:**

In the cohort study, ESBL-producing bacteria were isolated from 3 of 51 (5.9%) samples in the control group compared to 37 of 57 (89.5%) samples in the exposed group. A significant association was found between ESBL shedding and feeding raw pet food products (OR = 31.5). No other risk factors were identified in this study. ESBL-producing *Enterobacteriaceae* were isolated from 14 of 18 (77.8%) raw pet food products and 0 of 35 non-raw pet food products.

**Conclusions:**

This study shows a strong association between shedding of ESBL-producing bacteria in household cats and feeding raw pet food. Raw pet food was often contaminated with ESBL-producing *Enterobacteriaceae*.

## Introduction

Antimicrobial resistance poses a worldwide threat to human and animal health. Through inactivation of beta-lactam antibiotics (e.g. penicillins, 1^st^-4^th^ generation cephalosporins), extended-spectrum beta-lactamase (ESBL) and AmpC beta-lactamase (AmpC)-producing bacteria contribute to the increased risk of antibiotic treatment failures. Besides chromosomal spread, ESBL/AmpC-encoding genes are mainly transferred through mobile genetic elements, e.g. plasmids, increasing the rate of transmission. Thus far, ESBL/AmpC-producing *Enterobacteriaceae* (ESBL-pE) have been isolated from many sources including humans, food-producing animals, companion animals, wildlife, crops and environmental sources [1–7]. As distribution through the food-chain is a concern for human health, thorough investigation has been performed on ESBL-pE originating from food-producing animals [8–10]. However, direct contact with animals or their feces might also pose a risk to public health for transmission of antimicrobial resistant bacteria [11,12]. Therefore, companion animals living in close contact with their owners should be another focus point. Previous studies have found that canine and feline *E*. *coli* isolates possessed the same ESBL-encoding genes and were the same sequence types (STs) as those isolates from people [13,14]. Similar strains of ESBL-pE were found in dogs and their owners, suggesting household transmission [15,16]. Nevertheless, little is known about risk factors for ESBL/AmpC shedding in companion animals. Several risk factors for ESBL/AmpC shedding have been proposed, including hospitalization, antimicrobial treatment, and consumption of raw pet foods (RPF) [17–22]. Additionally, presence of coliforms in RPF, *Salmonella* as well as ESBL/AmpC-producing *E*. *coli*, has been shown [23–25]. Although feeding RPF has been proposed as a risk factor for ESBL/AmpC shedding before [20–22], and ESBL-pE have been found in RPF [25], an association has not been demonstrated yet. As the prevalence of ESBL-pE appears to be lower in cats than in dogs, feeding RPF may affect fecal shedding of ESBL-pE considerably in cats subjected to a raw diet [26].

To study raw pet food as a risk factor for ESBL/AmpC shedding in healthy household cats, (i) a cohort study was designed to investigate the association of fecal shedding of ESBL-pE with a raw meat diet and (ii) both raw and non-raw commercial pet foods were screened for the presence of ESBL-pE.

## Materials and methods

### Cohort study

A cohort study was set up at the Faculty of Veterinary Medicine, Utrecht University, Utrecht, the Netherlands, to investigate the association between shedding of ESBL-pE in household cats and a diet containing RPF. Participants were recruited through a pet owner database of a surveillance study conducted at the Faculty of Veterinary Medicine and through social media [27]. Three selection criteria were used. First, willingness of owners to fill out a questionnaire describing: the cat’s living environment, other in-contact animals, predation activities, health and diet, with an emphasis on feeding habits. Second, the ability to send in a weekly fecal sample for 3 consecutive weeks. Third, the ability to separate cats in a multiple cat household for sample collection, since only one cat per household was included. For cats to be enrolled in the exposed group, they had to be fed with RPF daily. All fecal samples were collected from the litter box and either sent to the laboratory by regular mail service (non-temperature controlled), or deposited at the institute by the cat owners. In accordance with the Dutch Animals Act (stb-2011-345; https://zoek.officielebekendmakingen.nl/stb-2011-345.html [in Dutch]) and European Union directive 2010/63/EC, the Animal Welfare Body Utrecht gave permission to proceed with this study without a project license, as the study design was observational, the cats remained in their own household with no alterations to their living conditions and animal sampling was not invasive.

### Pet food

To investigate RPF as a source of ESBL/AmpC shedding in household cats, various commercial RPF and non-raw pet food products (NRPF) were cultured for ESBL-pE. RPF products were defined as meat products not subjected to any form of processing e.g. dehydration, heating, high pressure treatment, except freezing and grinding. In contrast, NRPF products were dried, pasteurized, sterilized and/or dehydrated during the production process. A selection of available products was made based on frequently mentioned brands in the questionnaire. Different production batches of these products were obtained in local franchises of Dutch supermarkets, pet shops and garden centers in the Utrecht region of the Netherlands, purchased by the investigators or donated by cat owners. According to producer statements, all investigated pet food products were produced in accordance with European legislation (2011/142/EC, annex X) for pet foods. This regulation states that only animal by-products of category 3 materials (2009/1069/EC, article 10) may be used for production of animal protein. Such protein might include carcasses of animals that have been slaughtered in a slaughterhouse and were considered fit for human consumption following an ante-mortem inspection.

### ESBL bacteriology

A 1:10 (w/v) dilution of 0.5 grams of feces was suspended in 0.9% NaCl to inoculate 100 μl in 1 ml of LB-broth supplemented with 1 mg/L cefotaxime (LBC) (Oxoid, the Netherlands) for enrichment. The inoculated LB-broth was incubated overnight at 37°C. After incubation, 10 μl of LBC was streaked onto MacConkey agar plates supplemented with 1 mg/L cefotaxime (MCC) (Oxoid, the Netherlands) and incubated at 37°C overnight. Colonies cultured on MCC were designated as isolates with a non-wild type susceptibility to cefotaxime (nWT) [28]. When nWT isolates were found, a quantitative culture method (track dilution) [29] was used to estimate the number of nWT *Enterobacteriaceae* (detection limit was 10^2^ CFU/g feces) in a sample. Square MCC plates were inoculated with six 10-fold serial dilutions of the fecal suspension and cultured as previously described [21].

For each culture-positive MCC, lactose-fermenting colonies with typical *E*. *coli*-like colony morphology were chosen for further analysis. If *E*. *coli*-suspected colonies with multiple morphologies were present, one representative of each morphology was selected. Similarly, if lactose-non-fermenters were present, one colony representing each unique morphology was selected. The concentration (CFU/g) was calculated for each culture-positive plate, with a detection limit of 10^−2^ CFU/g feces for the enrichment step.

Frozen pet food products were processed for analysis immediately once they were thawed. Of each product, a sample of 25 grams was put in a stomacher bag with 225 ml LBC and homogenized for 5 minutes. The homogenized suspensions were incubated overnight at 37°C. Subsequently, 100 μl of the inoculated LBC was plated onto MCC and incubated overnight at 37°C. Three *E*. *coli*-like colonies were selected for further molecular analysis, preferably with different morphology. If more than three morphologies were observed, one colony representing each morphology was selected.

### Species identification

Matrix assisted laser desorption ionization-time of flight mass spectrometry (MALDI-TOF MS) (Bruker, Delft, the Netherlands) was used to identify the bacterial species of the obtained isolates.

### ESBL confirmation

Isolates were boiled for DNA extraction in 10 mM Tris-HCl, 1 mM disodium EDTA, pH 8.0 (Sigma-Aldrich, Switzerland). PCR and sequence analysis was done as previously described [21]. In short, conventional PCR was used to screen all lysates for presence of plasmid-mediated ESBL/AmpC gene families: *bla*_CTX-M_, *bla*_SHV,_ *bla*_OXA,_ *bla*_CMY,_ *bla*_TEM,_ and chromosomal *ampC*. All used primer combinations are listed in S1 Table. The PCR mix contained 5 μL DNA lysate, 2x GoTaq Hotstart Green Master Mix (Promega Benelux BV, Leiden, the Netherlands), 0.5 μM of each forward and reverse primers and molecular grade water. Purified PCR products (ExoSAP-IT; Affymetrix, Santa Clara, USA) were sent for sequence analysis (Baseclear, Leiden, the Netherlands) and analyzed using Bionumerics v7.1 (Applied Maths NV, Sint-Martens-Latem, Belgium). ESBL variants deposited at lahey.org/studies (last accessed 29-05-2016) were used as a reference.

### Multi locus sequence typing

*E*. *coli* isolates obtained from different consecutive fecal samples with similar ESBL genes, were further characterized with multi locus sequence typing (MLST) [30]. Sequences were compared to reference sequences provided on www.mlst.net (last accessed 29-05-2016) and analyzed using Bionumerics v7.5 (Applied Maths NV, Sint-Martens-Latem, Belgium).

### Statistical analysis

Risk factor analysis was performed using the GLIMMIX procedure in SAS version 9.4 (SAS Institute, Inc., Cary, NC, USA). Analysis was adjusted for clustering at cat level by treating this as random effect. Variables with a P-value < 0.3 in the univariate analysis were included in the multivariate analysis. A P-value < 0.05 was considered statistically significant.

## Results

### Cohort study

A total of 36 cats were included in the cohort study. The control group consisted of 17 cats and the exposed group consisted of 19 cats. As a part of their diet, cats in the exposed group were fed RPF daily. In the control group, no RPF was fed to the cats. All included cats were epidemiologically unrelated, living in different households, in various regions of the Netherlands. General characteristics of the cats enrolled in the cohort study are summarized in Table 1. A complete overview of the information obtained in the questionnaire is given in S2 Table. In total, 108 fecal samples were screened for the presence of ESBL-pE. In the control group, 3 of 51 samples were culture-positive on MacConkey agar plates supplemented with 1 mg/L cefotaxime (MCC). These samples originated from 3 different cats. In the exposed group, 37 of 57 samples were culture-positive; the positive samples were obtained from 17 cats (Table 2).

**Table 1:** General characteristics of the study population.

		Cohort	
		Control group	Exposed group
Total number of cats		17	19
Sex			
	Male	9 (53%)	8 (42%)
	Female	8 (47%)	11 (58%)
Age range		9 mo—16 yr Median 7.5 yr	9 mo—20 yr Median 8 yr
Outside			
	No	6 (35%)	7 (37%)
	Yes, every day	9 (53%)	10 (53%)
	Yes, less than every day	2 (12%)	2 (10%)
Eating prey			
	No	14 (82%)	11 (58%)
	Yes	3 (18%)	8 (42%)
Contact animals			
	None	6 (35%)	0
	Cats	3 (18%)	4 (21%)
	Dogs	5 (29%)	7 (37%)
	Both cats and dogs	3 (18%)	7 (37%)
	And other animals	3 (18%)	2 (10%)
	>2 contacts	4 (24%)	9 (47%)
Feed			
	Dry NRPF ^**a**^	8 (47%)	0
	Wet NRPF	0	0
	Dry and wet NRPF	9 (53%)	0
	Fresh RPF ^**a**^	0	0
	Frozen RPF	0	5 (26%)
	Fresh and frozen RPF	0	7 (37%)
	NRPF and RPF	0	7 (37%)
Health			
	No health problems	16 (94%)	16 (84%)
	AB use before study ^**a**^	1^b^ (6%)	2^d^ (10%)
	AB use during study	0	0
	Other meds before study	0	0
	Other meds during study	1^c^ (6%)	1^e^ (5%)

^a^ NRPF, non-raw pet food. RPF, raw pet food. AB, antibiotics.

^b^ This cat was treated with clavulanate-amoxicillin 3 months before start of the cohort study.

^c^ This cat was continuously treated with a corticosteroid drug, also during the cohort study.

^d^ These cats were treated with antibiotics before the start of the cohort study, the active ingredients are unknown.

^e^ This cat was treated on several occasions with a non-steroidal anti-inflammatory drug in the period of 6 months before the start of the cohort study and during the cohort study.

**Table 2:** ESBL and MLST characterization results of cohort study.

	Time points of weekly sample collection		
CatID ^a^	T1	T2	T3
C1	CTX-M-1 (1); CTX-M-1 + TEM-1b (1); CMY-2 (2)	N	N
C2	N	CTX-M-32 (1)	N
C3	CTX-M-1 (4)	N	N
C4-C17	N	N	N
E1	N	CTX-M-1 (3)	SHV-12 (2); CMY-2 + TEM-1b (2)
E2	CTX-M-1 (ST410) (2); CTX-M-2 + TEM-1b (1)	CTX-M-1 (ST410) (2)	CTX-M-1 (ST410) (2); CTX-M-32 (2)
E3	CTX-M-1 (3)	N	N
E4	CMY-2 (ST38) (2)	CTX-M-15 + TEM-1b (3)	CMY-2 (ST38) (3)
E5	N	N	CTX-M-1 (1); SHV-12 (2)
E6	N	CTX-M-1 (ST3381) (1); CTX-M-32 (1); SHV-12 (ST1850) (1)	CTX-M-1 (ST2607) (1); CTX-M-1 + TEM-1d (3); SHV-12 (ST2067) (1)
E7	CTX-M-1 + TEM-1b (ST58) (2)	CTX-M-1 + TEM-1b (ST58) (3)	CTX-M-1 + TEM-1b (ST58) (1)
E8	N	N	N
E9	N	N	CTX-M-1 (2); CMY-2 (1)
E10	N	CTX-M-14 (1)	CTX-M-1 + TEM-1b (3)
E11	CTX-M-2 + TEM-1b (3)	N	N
E12	CTX-M-1 + TEM-1b (ST69 and ST117) ^b^ (3)	CTX-M-1 + TEM-1b (ST69) (3)	CTX-M-1 + TEM-1b (ST69) (3)
E13	TEM-52c (3)	CTX-M-24 + TEM-1d (2); CMY-2 (1)	CTX-M-1 (2)
E14	N	CTX-M-1 (ST155) (1); CTX-M-1 + TEM-1b (1); CTX-M-32 (2); CTX-M-32 + TEM-1b (1); TEM-52c (Unknown ST) (1)	CTX-M-1 (ST398) (1); SHV-12 (1); TEM-52c (ST1284) (1)
E15	CMY-2 (1)	N	CTX-M-1 (1)
E16	CTX-M-1 (ST117) (2); CMY-2 (2)	N	CTX-M-1 (ST117) (1)
E17	CTX-M-1 + TEM-1b + OXA-1 (1); CTX-M-1 + TEM-1 + TEM-1b (Unknown ST) (1); CMY-2 (1)	CTX-M-1 (3)	CTX-M-1 + TEM-1 + TEM-1b (Unknown ST) (2); CTX-M-15 + TEM-1b + TEM-var ^c^ (1)
E18	CTX-M-1 (3); CMY-2 + TEM-32 (1)	CMY-2 (3)	SHV-12 (3)
E19	N	N	N

^a^ CatID: C(x) is cat in control group, E(x) is cat in exposed group. N, fecal sample without growth on MCC. Isolates with different ESBL types in one sample are separated by a semicolon. Number of isolates with ESBL type is given in brackets. *E*. *coli* ST is given when similar ESBL types were found in consecutive samples (underlined genes).

^b^ The same gene combination was found in two *E*. *coli* isolates with distinct STs obtained from the same fecal sample. ^c^ TEM-1var is a TEM-1 protein with an identical nucleotide sequence as deposited at GenBank GU371927.

CFU/g feces were determined for all culture-positive samples on MCC agar. In the control group, the three positive samples contained 2.88*10^4^ CFU/g feces, 2.00*10^5^ CFU/g feces and < 10^2^ CFU/g feces. In the exposed group, the mean CFU/g feces on MCC was 3.61*10^7^ CFU/g feces (9.43*10^3^–9.80*10^8^ CFU/g feces).

In total, 135 non-wild-type (nWT) *E*. *coli* isolates were identified. ESBL/AmpC-encoding genes were found in 114 *E*. *coli* isolates. *Bla*_CTX-M_ genes were found in 81 isolates, mostly of CTX-M group 1. Thirteen cats shed ESBL-pE in 2 or 3 samples during the study period (Table 2). Eight cats shed isolates possessing the same ESBL types in consecutive fecal samples. Of these 19 consecutive samples, 40 isolates were obtained and analyzed with MLST. Similar ESBL genes and *E*. *coli* STs were found in all three consecutive samples of cat E2, cat E7 and cat E12. Cat E4 and cat E16 also shed similar ESBL-producing *E*. *coli* STs, although not in consecutive samples. The ESBL/AmpC genes identified and MLST profiles are summarized in Table 2.

Univariate analysis on the data obtained through the questionnaires showed P-values < 0.3 for the variables ‘RPF’ (P-value < 0.0001), ‘contact animals’ (P-value = 0.04) and ‘prey’ (P-value = 0.24). In the multivariate analysis, only variable ‘RPF’ was significantly associated with shedding of ESBL-pE in feces (OR = 31.5; 95%CI: 5.2–192.1; P-value < 0.0003).

### Pet food

In total, 53 food products were screened. Eighteen different RPF products of 5 different brands, 15 dry non-RPF (NRPF) products of 7 different brands and 20 wet NRPF products of 8 different brands were screened for the presence of ESBL-pE. As described on the product packaging, poultry meat was used in 35 of all 53 products and beef was used in 19 products. No ESBL-pE were cultured from the NRPF products. Fourteen (77.8%) RPF products showed growth on MCC. These products were all frozen when purchased. The screening results for ESBL-pE from RPF are summarized in Table 3. ESBL/AmpC gene types found in these products were *bla*_CTX-M-1_, *bla*_CTX-M-3_, *bla*_CTX-M-15_, *bla*_CTX-M-32_, *bla*_CTX-M-2_, *bla*_CTX-M-14_, *bla*_CMY-2_ and *bla*_SHV-12_. *Bla*_CTX-M_ was the dominant ESBL gene type, often accompanied by a *bla*_TEM-1_ variant.

**Table 3:** ESBL characterization results for raw pet food products.

Product code ^a^	Number of tested product batches	Protein source	Product storage conditions	ESBL-producer identified	ESBL type
**A1**	1	Chicken (100%)	Frozen	Yes	CTX-M-32 + TEM-1b + TEM-1c; CMY-2
**A2**	2	Beef tripe (60%) and beef (20%)	Frozen	Yes	CTX-M-3 + TEM-1b; CTX-M-14 + TEM-1b; CTX-M-1
**A3**	2	Beef (100%)	Frozen	Yes	CTX-M-1; CTX-M-2 + TEM-1b; CTX-M-1 + TEM-1 + TEM-1b; CTX-M-32; CTX-M-2
**A4**	1	Beef (60%) and beef tripe (20%)	Frozen	Yes	CTX-M-15 + TEM-1b + TEM-var ^**b**^; CTX-M-32
**A5**	1	Beef tripe (100%)	Frozen	Yes	CTX-M-1 + TEM-1 + TEM-1b; CTX-M-32; CTX-M-32 + TEM-1b
**B1**	1	Beef and chicken	Frozen	Yes	CTX-M-15
**B2**	1	Duck	Frozen	Yes	CTX-M-1
**B3**	1	Lamb and chicken	Frozen	Yes	CTX-M-1 + TEM-1b
**B4**	1	Chicken and beef	Frozen	Yes	SHV-12; CMY-2 + TEM-1b
**C1**	1	Chicken, beef and mutton	Frozen	No	
**C2**	1	Lamb (30%)	Non-refrigerated	No	
**C3**	1	Chicken (30%)	Non-refrigerated	No	
**D1**	1	Duck	Frozen	Yes	CTX-M-1 + TEM-1c
**D2**	2	Chicken and beef	Frozen	Yes	CTX-M-1; CTX-M-32; CMY-2 + TEM-1b
**E1**	1	Chicken	Frozen	Yes	CMY-2
**E2**	1	Chicken	Frozen	Yes	CTX-M-1 + TEM-1 + OXA-1; CMY-2
**E3**	1	Duck	Frozen	No	
**E4**	1	Turkey	Frozen	Yes	CTX-M-1

^a^ Product code shows brand (A-E) and product (number). Percentage of protein sources were given if known. Different isolates are separated by a semicolon.Non-raw pet food product information is given in S3 Table.

^b^ TEM-1var is a TEM-1 protein with an identical nucleotide sequence as deposited at GenBank GU3719.

## Discussion

A cohort study in household cats was performed to investigate an association between feeding RPF and ESBL-pE shedding in companion animals. Furthermore, several raw and non-raw commercial pet foods were screened for presence of ESBL-pE.

In this study, 77.8% of all investigated RPF products showed contamination with viable ESBL-pE, while none of the NRPF products was contaminated with these bacteria. An earlier study by Nilsson *et al*. [25] found ESBL-pE in 23% samples of RPF. The combination of these observations with the high level of contamination found in this study, makes RPF a probable source for ESBL/AmpC shedding in companion animals. A strong association between feeding RPF and shedding of ESBL-pE in household cats was demonstrated in the cohort study (OR = 31.5). To our knowledge, this is the first time that an association of ESBL/AmpC shedding in companion animals with feeding RPF has been shown in a cohort study. No other risk factors for ESBL/AmpC shedding were identified in this study, possibly due to the limited sample size.

The most frequent ESBL/AmpC types found in RPF in this study were *bla*_CTX-M-1_, *bla*_CTX-M-15_, *bla*_CTX-M-2_, *bla*_CTX-M-14_, *bla*_CMY-2_, and *bla*_SHV-12_. As poultry meat and beef are the main protein sources for pet food products, it is not surprising that similar ESBL/AmpC types have previously been found in poultry, cattle and pigs [1]. ESBL/AmpC types found in the cohort study also resembled ESBL/AmpC types found in RPF, which strengthens the hypothesis of increased ESBL/AmpC shedding after consuming an ESBL-pE through a contaminated food source. Less frequent ESBL types found in this cohort study were *bla*_CTX-M-24,_ which was found in 2 isolates, and *bla*_CTX-M-32_ which was found in 7 isolates. These ESBL-types have been previously found in *E*. *coli* isolated from feline urine, wound and nasal samples [31–33].

Eleven exposed cats shed ESBL/AmpC-producing bacteria in consecutive samples. Since no information about fed RPF production batches was available, it is possible individual cats were fed with the same contaminated product throughout this study period. However, the probability of feeding several contaminated products consecutively is high, according to the high level of contamination of RPF products. Eight cats shed similar ESBL types in consecutive fecal samples. In 5 of these cats, consecutive identical combinations of ESBL type and *E*. *coli* ST were found. Three cats (E2, E7 and E12) continuously shed *E*. *coli* of the same ST possessing identical ESBL types. Continuous shedding may have occurred below the detection limit in additional animals. In most cats, frequent variation of present ESBL/AmpC types was seen during this short study period. Even cats shedding the same ESBL-pE continuously, carried additional ESBL/AmpC types that varied over time. The low persistence of ESBL types indicates that gut colonization with ESBL-pE is unlikely. This is supported by a similar frequent variation of ESBL types found in a longitudinal study in healthy household dogs [21]. Consistent exposure to ESBL-pE through contaminated feed and subsequent loss of ESBL-producing strains, seems to be a reasonable explanation for the observed ESBL/AmpC shedding in companion animals opposed to gut colonization.

This study shows the risk of feeding raw pet food to companion animals for both the animals as well as their owners handling raw pet food. Another possible route for ESBL uptake might be household transmission. Data on transmission of ESBL-pE between pets and humans is limited. Within-household sharing of ESBL-pE between humans and dogs was shown in 2 of 22 households in a Swedish study [15]. In both households, children < 3 years of age carried the ESBL-producing strain [15]. In another study, 4 isolate pairs of dogs and their owners were related as they showed identical PFGE patterns [16]. As dogs and owners were not sampled in the same time frame in these studies, actual transmission could not be established. Nevertheless, these studies support the possibility of within-household transmission of ESBL-pE, including human-to-pet transmission or vice versa.

Raw pet food products are shown to be an important risk factor for ESBL/AmpC shedding in household cats. Consistent exposure to raw pet food products seems to be accompanied by ESBL-pE shedding, opposed to gut colonization. Pet owners should be aware of a possible risk for ESBL uptake when handling raw pet food products.

## Supporting information

S1 Table Primer combinations for detection of ESBL-encoding genes. (DOCX)

S2 Table Overview of information obtained from questionnaire in cohort study. (XLSX)

S3 Table Pet food product information and results. (XLSX)
