# Supplementary material for: Raw pet food as a risk factor for shedding of extended-spectrum beta-lactamase-producing Enterobacteriaceae in household cats
Source: PLoS One. 2017 Nov 2;12(11):e0187239. doi: 10.1371/journal.pone.0187239 (PMC5667807; doi:10.1371/journal.pone.0187239)
Supplement: S1 Table — (DOCX) [file pone.0187239.s001.docx]

**Table S1. Primer combinations for detection of ESBL-encoding genes**

| Name | Gene | Sequence | Size product (bp) | Reference |
| --- | --- | --- | --- | --- |
| CTX-M-F | CTX-M-gr25 | ATG TGC AGY ACC AGT AAR GTK ATG GC |  | (1) |
| CTX-M-R | CTX-M-gr25 | TGG GTR AAR TAR GTS ACC AGA AYS AGC GG | 592 |  |
| CTX-M-1g Fw | CTX-M-gr1 | CCC ATG GTT AAA AAA TCA CTG C |  | (2) |
| CTX-M-1g Rv | CTX-M-gr1 | CAG CGC TTT TGC CGT CTA AG | ~1000 |  |
| CTX-M-2-F | CTX-M-gr2 | ATG ATG ACT CAG AGC ATT CG |  | (3) |
| CTX-M-2-R | CTX-M-gr2 | TGG GTT ACG ATT TTC GCC GC | 865 |  |
| CTX-M-9F | CTX-M-gr9 | TGG TGA CAA AGA GAG TGC AAC G |  | (4) |
| CTX-M-9R | CTX-M-gr9 | TCA CAG CCC TTC GGC GAT | 874 |  |
| CTX-M-9_792_F | CTX-M-14-like | CTA TTT TAC CCA GCC GCA AC |  | (5) |
| CTX-M-9_1029_R | CTX-M-14-like | GTT ATG GAG CCA CGG TTG AT | 238 |  |
| TEM-F | TEM | GCG GAA CCC CTA TTT G |  | (1) |
| TEM-R | TEM | ACC AAT GCT TAA TCA GTG AG | 964 |  |
| TEM-seq | TEM | GCC AAC TTA CTT CTG ACA ACG |  | (6) |
| SHV-F | SHV | TTA TCT CCC TGT TAG CCA CC |  | (1) |
| SHV-R | SHV | GAT TTG CTG ATT TCG CTC GG | 795 |  |
| CMY-2-F | CMY | ATG ATG AAA AAA TCG TTA TGC TGC |  | (1) |
| CMY-2-R | CMY | GCT TTT CAA GAA TGC GCC AGG | 1117 |  |
| CMY-F-838 | CMY | TGG CGT ATT GGC GAT ATG TA |  | (6) |
| CMY-R-857 | CMY | TAC ATA TCG CCA ATA CGC CA |  |  |
| O1-GD2M-F | OXA-1-like | CAA CGG ATT AAC AGA AGC ATG GCT CG |  | (7) |
| O1-GD2M-R | OXA-1-like | GCT GTR AAT CCT GCA CCA GTT TTC CC | 194 |  |
| O2-GD2M-F | OXA-2-like | GAC CAA GAT TTG CGA TCA GCA ATG CG |  | (7) |
| O2-GD2M-R | OXA-2-like | CYT TGA CCA AGC GCT GAT GTT CYA CC | 254 |  |
| O10-GDM-F | OXA-10-like | CGC CAG AGA AGT TGG CGA AGT AAG |  | (7) |
| O10-GDM-R | OXA-10-like | GAA ACT CCA CTT GAT TAA CTG CGG | 138 |  |
| OXA-48A | OXA-48-like | TTG GTG GCA TCG ATT ATC GG |  | (8) |
| OXA-48B | OXA-48-like | GAG CAC TTC TTT TGT GAT GGC | 743 |  |
| O23-GDM-F | OXA-23-like | CCT GAT CGG ATT GGA GAA CCA G |  | (7) |
| O23-GDM-R | OXA-23-like | GAT GCC GGC ATT TCT GAC CG | 512 |  |
| O24-GDM-F | OXA-24-like | GGT CGA TAA TTT TTG GTT AGT TGG CCC |  | (7) |
| O24-GDM-R | OXA-24-like | CCA TTA GCT TGC TCC ACC CAA CCA G | 236 |  |
| O51-GDM-F | OXA-51-like | GAC CGA GTA TGT ACC TGC TTC GAC C |  | (7) |
| O51-GDM-R | OXA-51-like | GAG GCT GAA CAA CCC ATC CAG TTA ACC | 493 |  |
| O58-GDM-F | OXA-58-like | GTG CTG AGC ATA GTA TGA GTC GAG C |  | (7) |
| O58-GDM-R | OXA-58-like | GGT CTA CAG CCA TTC CCC AGC C | 629 |  |
| *amp*C1-_71_ | Amp-Chromosomal | AAT GGG TTT TCT ACG GTC TG |  | (9) |
| *amp*C2-_120_ | Amp-Chromosomal | GGG CAG CAA ATG TGG AGC AA | 191 |  |

**References supplemental material**

1. Dierikx C, van Essen-Zandbergen A, Veldman K, Smith H, Mevius D. Increased detection of extended spectrum beta-lactamase producing *Salmonella enterica* and *Escherichia coli* isolates from poultry. Vet Microbiol. Elsevier B.V.; 2010 Oct 26;145(3–4):273–8.

2. Carattoli A, García-Fernández A, Varesi P, Fortini D, Gerardi S, Penni A, et al. Molecular epidemiology of *Escherichia coli* producing extended-spectrum beta-lactamases isolated in Rome, Italy. J Clin Microbiol. 2008 Jan 1;46(1):103–8.

3. Jiang X, Zhang Z, Li M, Zhou D, Ruan F, Lu Y. Detection of extended-spectrum beta-lactamases in clinical isolates of *Pseudomonas aeruginosa*. Antimicrob Agents Chemother. 2006 Sep;50(9):2990–5.

4. Paauw A, Fluit AC, Verhoef J, Leverstein-van Hall MA. *Enterobacter cloacae* outbreak and emergence of quinolone resistance gene in Dutch hospital. Emerg Infect Dis. 2006 May;12(5):807–12.

5. Dierikx CM, van Duijkeren E, Schoormans AHW, van Essen-Zandbergen A, Veldman K, Kant A, et al. Occurrence and characteristics of extended-spectrum-β-lactamase- and AmpC-producing clinical isolates derived from companion animals and horses. J Antimicrob Chemother. 2012 Jun;67(6):1368–74.

6. Hordijk J, Mevius DJ, Kant A, Bos MEH, Graveland H, Bosman AB, et al. Within-farm dynamics of ESBL/AmpC-producing *Escherichia coli* in veal calves: a longitudinal approach. J Antimicrob Chemother. 2013 Nov;68(11):2468–76.

7. Voets GM, Fluit AC, Scharringa J, Cohen Stuart J, Leverstein-van Hall MA. A set of multiplex PCRs for genotypic detection of extended-spectrum β-lactamases, carbapenemases, plasmid-mediated AmpC β-lactamases and OXA β-lactamases. Int J Antimicrob Agents. Elsevier B.V.; 2011 Apr;37(4):356–9.

8. Aubert D, Naas T, Héritier C, Poirel L, Nordmann P. Functional characterization of IS1999, an IS4 family element involved in mobilization and expression of beta-lactam resistance genes. J Bacteriol. 2006 Sep;188(18):6506–14.

9. Caroff N, Espaze E, Bérard I, Richet H, Reynaud A. Mutations in the ampC promoter of *Escherichia coli* isolates resistant to oxyiminocephalosporins without extended spectrum ß-lactamase production. FEMS Microbiol Lett. 1999 Apr;173(2):459–65.
